# Increased motor cortex excitability during motor imagery in brain-computer interface trained subjects

**DOI:** 10.3389/fncom.2013.00168

**Published:** 2013-11-22

**Authors:** Olesya A. Mokienko, Alexander V. Chervyakov, Sofia N. Kulikova, Pavel D. Bobrov, Liudmila A. Chernikova, Alexander A. Frolov, Mikhail A. Piradov

**Affiliations:** ^1^Research Center of Neurology Russian Academy of Medical ScienceMoscow, Russia; ^2^Institute of Higher Nervous Activity and Neurophysiology of RASMoscow, Russia; ^3^Technical University of OstravaOstrava, Czech Republic

**Keywords:** brain-computer interface, motor imagery, navigated TMS, functional MRI, neurorehabilitation

## Abstract

**Background**: Motor imagery (MI) is the mental performance of movement without muscle activity. It is generally accepted that MI and motor performance have similar physiological mechanisms.

**Purpose**: To investigate the activity and excitability of cortical motor areas during MI in subjects who were previously trained with an MI-based brain-computer interface (BCI).

**Subjects and Methods**: Eleven healthy volunteers without neurological impairments (mean age, 36 years; range: 24–68 years) were either trained with an MI-based BCI (BCI-trained, *n* = 5) or received no BCI training (*n* = 6, controls). Subjects imagined grasping in a blocked paradigm task with alternating rest and task periods. For evaluating the activity and excitability of cortical motor areas we used functional MRI and navigated transcranial magnetic stimulation (nTMS).

**Results**: fMRI revealed activation in Brodmann areas 3 and 6, the cerebellum, and the thalamus during MI in all subjects. The primary motor cortex was activated only in BCI-trained subjects. The associative zones of activation were larger in non-trained subjects. During MI, motor evoked potentials recorded from two of the three targeted muscles were significantly higher only in BCI-trained subjects. The motor threshold decreased (median = 17%) during MI, which was also observed only in BCI-trained subjects.

**Conclusion**: Previous BCI training increased motor cortex excitability during MI. These data may help to improve BCI applications, including rehabilitation of patients with cerebral palsy.

## Highlights

- The cerebellum, thalamus, and Brodmann areas 3 and 6 were activated during motor imagery.
- The primary motor cortex was activated in BCI-trained subjects and not in controls.
- TMS motor threshold was decreased by 6–18% (median, 17%) during motor imagery in BCI-trained subjects.
- In BCI-trained subjects, evoked motor responses were larger during motor imagery than at rest.

## Introduction

Modularity is important concept in understanding mechanisms of motor control and motor learning. Recent investigations of muscle synergies, motor primitives, compositionality, basic action concepts, and related work in machine learning have contributed to advance our understanding of the architecture underlying rich motor behaviors (Lacquaniti et al., 2013). One of the most interesting topic in researches of motor system modularity and organization is study of motor imagery and changes in neural networks during it.

Motor imagery (MI) activates brain regions that participate in motor control (Crammond, 1997; Jeannerod, 2001; Stippich et al., 2002; Ehrsson et al., 2003; Neuper et al., 2005). These structures include the premotor and supplementary motor cortices (Brodmann area 6), parietal cortical areas, cingulate gyrus, basal ganglia, and cerebellum. The primary motor cortex (Brodmann area 4) is also active during MI (Jeannerod, 2001; Neuper et al., 2005; Sharma et al., 2006; Dickstein and Deutsch, 2007; Mulder, 2007). Furthermore, several studies using transcranial magnetic stimulation (TMS) demonstrated increased corticospinal excitability and increased amplitudes of motor evoked potentials (MEPs) during MI (Fadiga et al., 1999; Hashimoto and Rothwell, 1999; Vargas et al., 2004; Cicinelli et al., 2006; Stinear et al., 2006; Pichiorri et al., 2011).

These previous findings led scientists to develop an MI training paradigm to stimulate neuroplastic changes in patients with paresis resulting from brain injury, or for use in athletic training. An electroencephalography (EEG)-based brain-computer interface (BCI) is a promising method to support MI during such training. The BCI transforms EEG signals generated during MI into commands that can control an external device (Prasad et al., 2010; Mokienko and Chernikova, 2011; Shih et al., 2012). Modulation of the sensorimotor rhythm can serve as the signal of brain activity during MI (Pfurtscheller and Lopes da Silva, 1999). However, the plasticity-related changes resulting from BCI-supported MI training have not been studied in detail. Furthermore, no study has included the navigated transcranial magnetic stimulation (nTMS) method, or compared functional magnetic resonance imaging (fMRI) and nTMS data for functional mapping during MI in BCI-trained and not trained subjects. The aim of our experiment was to investigate the activity and excitability of different cortical motor areas during MI in BCI-trained and BCI-naïve subjects.

## Materials and methods

### Participants

The inclusion criteria were an age of 20–70 years old, an absence of neurological disorders, normal vision, right-handedness, and written informed consent. Eleven volunteers (mean age, 36 years; age range, 24–68 years; 7 males and 4 females) were included into the study. Subjects of group 1 (*n* = 5, mean age = 45.8 y.o) had 10 to 15 sessions of BCI-supported training 20–30 min each. The training course was followed by fMRI and nTMS examinations. Subjects of group 2 (*n* = 6, mean age = 27.6 y.o) were tested without preliminary training session. All subjects underwent fMRI and nTMS after the training sessions. The protocol was approved by the local Ethical Committee of the Research Center of Neurology of RAMS, Moscow. All subjects provided written informed consent.

### BCI training

The BCI training was based on EEG activity patterns recording during grasping MI. Subjects sat comfortably in an armchair located 1 m from a computer screen that presented visual instructions. Subjects visually fixated on a circle presented in the center of the screen and received instructions from three surrounding figures (rhomboidal arrows). Subjects were given three commands instructing them to relax (upper arrows were illuminated) or imagine slow grasping movements with the right or left hand (right or left arrow illuminated). The “Relax” command meant that the subject had to sit still and look at the center of the screen. Commands were presented randomly, each of 10-s duration. For each subject, training was performed in 10–15 experimental days, with one 20–30 min session performed each day. Intervals between training sessions were 1–4 days.

A visual cue provided the subject with feedback regarding the mental task recognition: the central circle turned green if the classifier recognized the task in agreement with the given command, or remained white if the signal was not recognized. The EEG was registered with 30 electrodes distributed over the head in accordance with the standard international 10–20 system. EEG signals were filtered from 5–30 Hz. We used a Bayesian approach for EEG pattern classifying. The activity sources most relevant for BCI functioning were identified using an independent component analysis (ICA). Classification accuracy was measured with Cohen's kappa, a parameter conventionally used in BCI studies (Kohavi and Provost, 1998). A kappa of 1 indicates perfect recognition, whereas a kappa of 0 indicates random recognition.

### fMRI

fMRI was conducted using a Magnetom Avanto 1.5-T MRI system (Siemens, Germany). Standard axial T2-weighted turbo-spin echo imaging was performed initially to rule out pathological changes in brain tissue [repetition time (TR), 4000 ms; echo time (TE), 106 ms; section thickness, 5.0 mm; matrix, 230 × 230 mm; imaging time, 2 min 2 s]. Anatomical data were obtained with sagittal T1-weighted gradient echo imaging with isometric voxels (Ò1 multiplanar reconstruction: TR, 1940 ms; TE, 3.1 ms; TI, 1100 ms; section thickness, 1.0 mm; matrix, 256 × 256 mm; imaging time, 4 min 23 s). During the fMRI experiment, subjects performed the same task that was performed during the BCI training sessions, but without feedback. For each subject, three sets of functional data were obtained representing different conditions, including rest (8 replicates) and right or left hand movement imagery (4 replicates each). The imaging mode used was axial T2^*^ gradient echo (TR, 3800 ms; TE, 50 ms; matrix, 192 × 192 mm, section thickness, 3 mm) with fat suppression and correction for motion. The imaging time was 6 min 10 s.

The data analysis was performed in the MATLAB (Mathworks, Natick, MA, USA) environment using the statistical package for processing in SPM8 (Welcome Trust Center of Neuroimaging, London, UK). The first step of analysis corrected head movement artifacts. Next, the functional data were translated to Montreal Neurological Institute (MNI) coordinates (i.e., normalization). Standard MNI coordinates, which are used in the SPM8 package, were developed and adopted by the International Consortium for Brain Mapping. In the next step, the normalized data were smoothed. This step was followed by a classic analysis that used generalized linear models. The results from each subject were used in the group analysis to identify areas showing task-specific activity.

### nTMS

Neurophysiological investigation was performed using nTMS with the NBS eXimia Nexstim apparatus (Finland). It includes 70 mm figure-eight-shaped BiPulse Nexstim coil, with a maximal magnetic field strength of 199 V/m and a magnetic impulse duration of 280 μs. The coil was placed anteromedially at a 45° angle from the midline. The stimulated hemisphere was not the same for all the subjects and was chosen randomly.

As a first step, all subjects underwent an MRI investigation on a Magnetom Symphony 1.5 T scanner (Siemens, Germany) using a T1 multiplanar reconstruction regime (MPR); the data were loaded into the NBS eXimia Nexstim system to obtain subjects' individual 3D brain models. Following that, real anatomical entities were matched to their MRI representations.

The MEPs were recorded using a standard EMG machine (Nexstim, EMD, Finland) and surface electrodes. MEPs were recorded by placing 0.6 cm^2^ EMG electrodes on the target muscle being mapped [abductor pollicis brevis (APB), flexor carpi ulnaris (FCU), and extensor carpi radialis (ECR)] which were positioned according to the atlas of Leis and Trapani (2000), according to the belly–tendon principle. The ground electrode was placed on the right clavicle or on the upper third of the right forearm. We then determined the resting motor threshold (MT), defined as the lowest stimulation intensity allowing evocation of motor responses 0.50 mV peak to peak amplitude in 5/10 trials with the patient at rest (Rossini et al., 1994). Resting MT was measured in present (%) of the maximum intensity of the magnetic stimulator (1,5 Tesla). Evoked motor responses (EMRs) and their amplitudes and latencies were recorded for each target muscle. Cortical motor representations were constructed from these observations.

In the first step of the experiment, the areas of interest (contralateral primary motor and premotor cortices) were stimulated with magnetic fields of 80–110 V/m to identify EMRs with amplitudes of 100–500 μV. The resting MT was determined for each site by the largest detected EMR amplitude. Cortical representations of the target muscles were mapped at 110% intensity of the determined resting MT. The mean EMR amplitudes and muscle motor representations were evaluated during cortical mapping.

In the second step of the experiment, the passive EMR threshold was determined and the motor representations were mapped while subjects imagined grasping with the contralateral hand. The motor representations were mapped using stimulus intensities as in the first step. The hand was positioned on the armrest in the neutral position of the radiocarpal joint.

Statistical analysis of quantifiable data was performed using a repeated measures analysis of variance (ANOVA) and Newman-Keuls *post-hoc* test using the Statistica 6.0 software package (StatSoft, 2003). The data are presented as the median and 25–75% quartiles. Differences were considered significant at *p* < 0.05.

## Results

### Subjects

Eleven volunteers (mean age, 36 years; age range, 24–68 years; 7 men, 4 women) participated in the study. Subjects in group 1 (*n* = 5) underwent 10 to 15 sessions of BCI-supported training that were each 20–30 min in duration. The training course was followed by fMRI and nTMS examinations. Subjects in group 2 (*n* = 6) were tested without performing preliminary training sessions.

### BCI training

The achieved accuracy rates (median Cohen's kappa) were 0.46 [0.45; 0.52]. BCI control for all subjects was achieved with sensorimotor rhythm modulation. MI was accompanied with desynchronization of mu and low beta rhythms (i.e., event-related desynchronization) (Pfurtscheller and Lopes da Silva, 1999). This signal was a recognizable command for the BCI.

### Functional MRI

**Figure 1:**
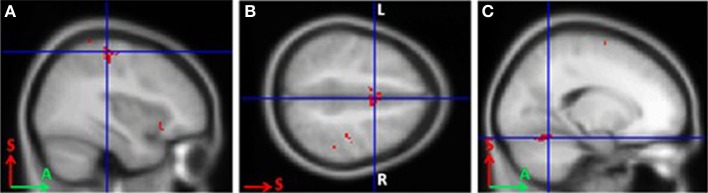
**Areas of activation during grasping imagery in BCI-trained subjects (group analysis of fMRI data, Left hand imagery > Rest, *p* < 0.0005). (A)**, Brodmann areas 3 and 4; **(B)**, supplementary motor cortex; **(C)**, cerebellum.

In BCI-trained subjects, MI was accompanied by activity in the contralateral somatosensory (Brodmann area 3), primary motor (Brodmann area 4), and premotor cortical areas. Activity also was observed in the bilateral supplementary motor cortex (Brodmann area 6), contralateral ventral lateral nucleus of thalamus, and ipsilateral cerebellum (*p* < 0.0005; Figure 1).

**Figure 2:**
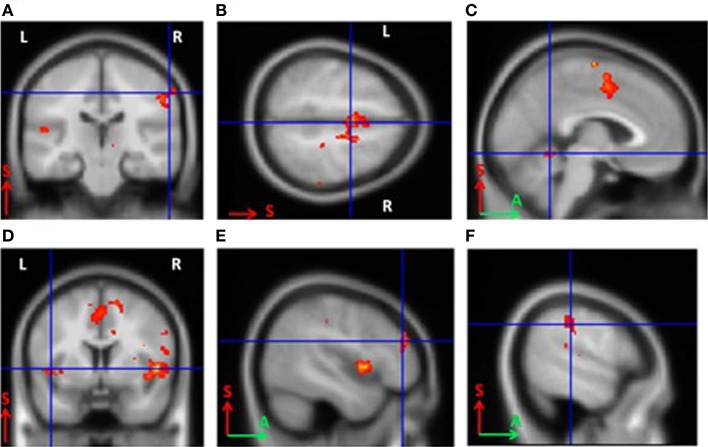
**Areas of activation during grasping imagery in untrained subjects (group analysis of fMRI data, Left hand imagery > Rest, *p* < 0.0005). (A)**, Brodmann areas 3 and 4; **(B)**, supplementary motor cortex; **(C)**, cerebellum; **(D)**, insula; **(E)**, Brodmann area 9; **(F)**, Brodmann area 40.

For BCI-naïve subjects, activity was observed in the contralateral somatosensory (Brodmann area 3) and premotor cortical areas, as well as the supplementary motor cortex (Brodmann area 6) bilaterally. Other activated areas included the contralateral ventral lateral nucleus of the thalamus, ipsilateral cerebellum, contralateral Brodmann area 9, and bilateral Brodmann areas 40 and 13 (*p* < 0.0005). The primary motor cortex was not activated in untrained subjects (Figure 2). The areas of activation including the somatosensory, premotor, and supplementary motor areas observed during MI were significantly larger in BCI-naïve subjects than in BCI-trained subjects (*p* < 0.01).

### nTMS

In BCI-trained subjects, the passive MT for the motor cortex decreased by 6–18% (median change was 17%) during MI compared to the rest condition. In BCI-naïve subjects, the threshold change during MI compared to rest were not significant and were inconsistent among subjects. The threshold decreased by 1–8% in three subjects, insignificantly increased in two subjects, and was unchanged in one subject. The MT changes were statistically significant only for BCI-trained subjects (*p* < 0.01, Table 1).

**Table 1:** **Motor thresholds and evoked motor responses during rest and motor imagery for the two groups (represented as median, [25th and 75th percentiles])**.

	**Group 1 (BCI-trained)**	**Group 2 (untrained)**
Motor threshold, rest	64.0 [59.0; 67.0]	55.5 [45.0; 69.0]
Motor threshold, motor imagery	53.0 [49.0; 58.0]	52.0 [45.0; 69.0]
*P*	***<0.01***	0.51
EMR APB, rest (μV)	216.4 [131.3; 315.9]	272.2 [175.4; 544.0]
EMR APB, motor imagery (μV)	365.1 [240.6; 515.2]	232.7 [158.5; 293.8]
*P*	***0.03***	0.24
EMR FCU, rest (μV)	170.7 [150.1; 280.5]	257.3 [186.4; 308.8]
EMR FCU, motor imagery (μV)	267.2 [254.5; 487.1]	280.2 [223.1; 351.6]
*P*	0.09	0.23
EMR ECR, rest (μV)	221.3 [213.7; 301.6]	266.2 [202.1; 437.2]
EMR ECR, motor imagery (μV)	433.3 [405.6; 809.7]	280.1 [188.7; 491.8]
*P*	***0.01***	0.89

EMR, evoked motor response; APB, abductor pollicis brevis; FCU, flexor carpi ulnaris; ECR, extensor carpi radialis. ANOVA with Newman-Keuls post-hoc test.

For APB, the median change in motor response during MI compared to rest condition was 63% in BCI-trained subjects, and 11% in BCI-naïve subjects. For ECR, the change was 150% in BCI-trained subjects and 1% in BCI-naïve subjects. In BCI-trained subjects, the responses in APB and ECR (mean EMR) during MI were significantly higher during MI compared to the rest condition (APB, *p* = 0.03; ECR, *p* = 0.01). In BCI-naïve subjects, the differences in EMR were not significant (APB, *p* = 0.24; ECR, *p* = 0.23, Table 1).

**Figure 3:**
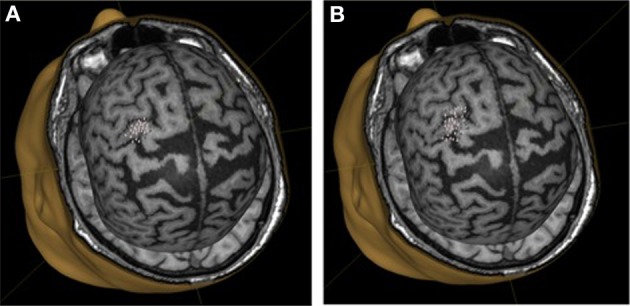
**Motor representation of target muscles in a BCI-trained subject. (A)**, background mapping; **(B)**, mapping during motor imagery.

**Figure 4:**
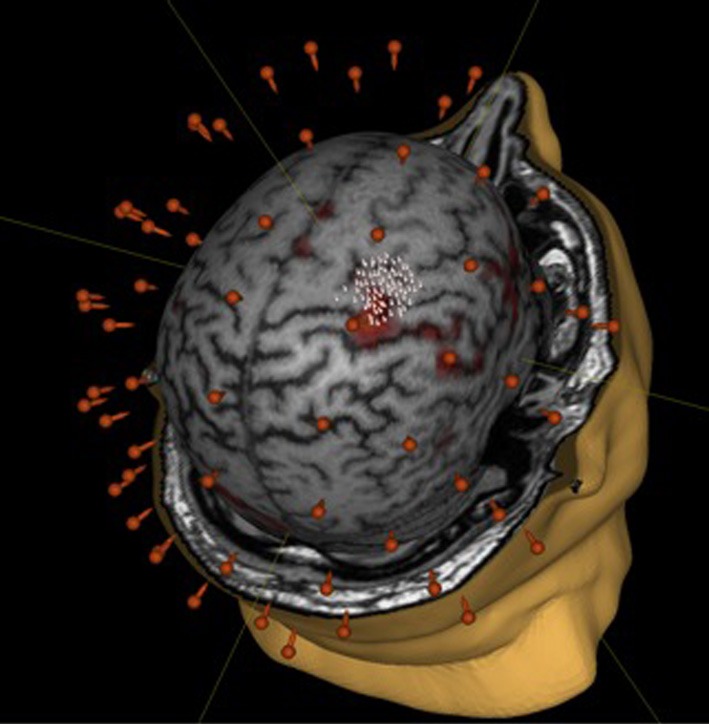
**Comparison of mapping results obtained during motor imagery in a BCI-trained subject by using fMRI (fist clenching imagery task) and nTMS**.

We did not observe statistically significant increases in mean EMR amplitude in FCU for either group (Table 1). The median change in motor response was 78% in BCI-trained subjects and 12% in BCI-naïve subjects. Moreover, in BCI-trained subjects, stimulation induced EMRs that were larger during MI than at rest, which was not observed in BCI-naïve subjects (Figure 3). A comparison of nTMS and fMRI maps revealed partial overlap of motor areas detected by these two methods (Figure 4).

## Discussion

The activation and excitability of the motor cortex during MI is different in BCI-trained and BCI-naïve subjects, and this difference can be detected with a combination of fMRI and nTMS.

### MI and activation of motor structures

The fMRI analysis revealed the same brain areas were active during MI for both groups. These areas included the contralateral somatosensory, contralateral premotor, supplementary motor cortex bilaterally, contralateral ventral lateral nucleus of thalamus, and ipsilateral cerebellum. Similar activation patterns were described in other fMRI-based MI studies (Jeannerod, 2001; Neuper et al., 2005; Sharma et al., 2006; Dickstein and Deutsch, 2007; Mulder, 2007).

In the literature, there is a debate regarding what role the primary motor cortex plays in MI, as some studies failed to observe its activation (Parsons et al., 1995; Hanakawa et al., 2003; Meister et al., 2004; de Lange et al., 2005). In our study, primary motor cortex activation was observed only in BCI-trained subjects. Therefore, we suppose primary motor cortex is involved in MI in individuals who can “successfully” imagine a movement, or who have been trained to do so (e.g., BCI-supported training).

The somatosensory, premotor, and supplementary motor cortical areas were activated during MI, and were larger on fMRI in BCI-naïve subjects. This is in agreement with the principles implying localization of new or skilled movements.

In BCI-naïve subjects, we observed bilateral activation of Brodmann area 40, the complex associative cortex. This region plays a central role in developing cognitive strategies and motor programs, and its activation was described in several previous MI studies (Gerardin et al., 2000; Lafleur et al., 2002; Jackson et al., 2003). This associative area was reported to be activated predominantly in the left hemisphere during complex motor performed by right-handed individuals (Gerardin et al., 2000). In addition, the dorsolateral prefrontal cortex (Brodmann area 9) was active in BCI-naïve subjects. This associative area represents the highest level of motor planning and regulation, and plays an important role in sensory and mnemonic information integration and working memory processes (Derrfuss et al., 2004). Right and left insula activation can be associated with cognitive control, task coordination, and working memory involvement (Derrfuss et al., 2004). It should be noted that BCI-trained subjects did not show significant activity in the associative areas.

### MI and corticospinal excitability

Our nTMS findings indicate that MI is generally associated with a decrease in the evoked response threshold, an increase in the EMR amplitude, and an expansion of evoked response areas against the background of decreased excitation thresholds. Together, these changes reflect increased motor cortex excitability during MI. These changes are increased with MI training and often do not occur in untrained individuals. Our results are in agreement with the findings of other studies using classical TMS (without MRI navigation) (Fadiga et al., 1999; Hashimoto and Rothwell, 1999; Vargas et al., 2004; Cicinelli et al., 2006; Stinear et al., 2006).

### nTMS and investigation of MI

Pichiorri et al. (2011) used TMS to assess the neuroplastic changes associated with MI-based BCI training. In that study, 10 healthy volunteers participated in 6–8 40-min BCI sessions. The training resulted in a significant increase in motor cortex excitability, and enhanced EMRs in target muscles during MI (Pichiorri et al., 2011). The TMS used in those studies was not navigated using MRI or fMRI data. In contrast to conventional TMS, nTMS allows local and precise stimulation based on an individual's MRI data (Chervyakov et al., 2013). This technique makes it possible to assess cortical excitability with a high spatial (2 mm) and temporal resolution. In the present study, we obtained similar results in terms of EMR, but we used both nTMS and fMRI. nTMS allowed us to map target muscle representations during MI for each subject based on MRI and fMRI data.

nTMS can be used to evaluate the dynamics of neuroplastic processes accompanying MI. MI mapping is most commonly performed with fMRI. In this case, the indirect measure of brain activity is the BOLD signal. The main advantage of fMRI is its high spatial resolution of approximately 1 mm (deCharms et al., 2004). However, the temporal resolution of this technique is relatively low, reaching 1–2 s. In addition, the physiological slowing of the hemodynamic response ranges from 3–6 s (Weiskopf et al., 2004). Most fMRI-based studies of MI do not involve EMG activity. In contrast, TMS provides a high temporal and spatial resolution. Surface EMG recording during TMS mapping makes it possible to control for the lack of muscle control during MI. Moreover, this mapping technique is based on directed and selective cortex stimulation, whereas in fMRI mapping, brain activity is evaluated based on an indirect signal.

### Comparison of fMRI and nTMS motor representation maps

The activity foci determined by the group analysis of fMRI data were in agreement with previously published data from other MI studies (Jeannerod, 2001; Neuper et al., 2005; Sharma et al., 2006; Dickstein and Deutsch, 2007; Mulder, 2007; Pichiorri et al., 2011). The discrepancy between the results for motor area mapping obtained using the two techniques (fMRI and nTMS) can be explained by the fact that TMS has a direct and selective effect on corticospinal pathways, whereas fMRI reflects BOLD signal changes associated with task performance (i.e., MI). A large study aimed at comparing these two neuroimaging techniques showed the distance between motor areas identified by fMRI and nTMS ranged from 0–21.7 mm (3.70 ± 4.85 mm) (Neuvonen and Niskanen, 2009).

### MI training and its clinical application in neurological rehabilitation

Changes in EMR amplitudes and cortical representations were mainly associated with a decrease in motor thresholds in individuals who had undergone MI training in a similar task. The EMR threshold reflects motor cortical excitability and was shown to be an informative parameter in several neurological diseases (Nikitin and Kurenkov, 2003). Our results suggest that MI training has a significant effect on cortical motor representations, which is probably comparable to that of actual motor training. Therefore, MI can be recommended as a rehabilitation practice in patients with severe motor deficiencies resulting from central nervous system injury.

To conclude, although the number of participants in this experiment was small, the results suggest the possibility of appropriate and optimal neuroplasticity control using BCI training. The considerations discussed above also suggest that nTMS is a highly promising method for investigating neurological plasticity. Nevertheless, further combined TMS-MRI-fMRI studies are required to determine its optimal application sites.

### Conflict of interest statement

The authors declare that the research was conducted in the absence of any commercial or financial relationships that could be construed as a potential conflict of interest.
